# Declining trend in transmitted drug resistance detected in a prospective cohort study of acute HIV infection in Bangkok, Thailand

**DOI:** 10.7448/IAS.19.1.20966

**Published:** 2016-10-31

**Authors:** Donn J Colby, Trevor A Crowell, Sunee Sirivichayakul, Suteeraporn Pinyakorn, Eugene Kroon, Khunthalee Benjapornpong, Jintana Intasan, Rapee Trichavaroj, Sodsai Tovanabutra, Merlin Robb, Praphan Phanuphak, Jintanat Ananworanich, Nittaya Phanuphak, on behalf of the Search 010/RV 254 Study Group

**Affiliations:** 1 SEARCH-Thailand, Bangkok, Thailand; 2 The Thai Red Cross AIDS Research Centre, Bangkok, Thailand; 3 U.S. Military HIV Research Program, Walter Reed Army Institute of Research, Silver Spring, MD, USA; 4 Henry M. Jackson Foundation for the Advancement of Military Medicine, Bethesda, MD, USA; 5 Department of Medicine, Faculty of Medicine, Chulalongkorn University, Bangkok, Thailand; 6 Department of Retrovirology, Armed Forces Research Institute of Medical Sciences – US Component, Bangkok, Thailand

**Keywords:** transmitted drug resistance, acute HIV infection, men who have sex with men, Thailand

## Abstract

**Introduction:**

As availability of antiretroviral therapy expands in developing countries, the risk for transmission of drug-resistant HIV also increases. Patients with acute HIV infection (AHI) provide an opportunity for real-time monitoring of transmitted drug resistance (TDR). SEARCH 010/RV 254 study is a prospective, longitudinal study of AHI. This analysis was performed to characterize changes in TDR over time in persons enrolled in the AHI cohort.

**Methods:**

Genotype testing for TDR mutations was performed on 229 subjects enrolled from 2009 to 2014.

**Results:**

The cohort was predominantly male (95%) and men who have sex with men (92%). TDR prevalence was 7.0%, declining from 12.5% in 2009–2010 to 4.8% in 2013–2014 (*p*=0.08). By drug class, resistance prevalence was 3.6% for proteases inhibitors, 2.6% for nucleoside/nucleotide reverse transcriptase inhibitors and 2.2% for non-nucleoside reverse transcriptase inhibitors. The greatest decline in prevalence was seen in the non-nucleoside reverses transcriptase inhibitors, from 9.4% in 2009–2010 to 0.7% in 2013–2014 (*p*=0.005).

**Conclusions:**

TDR appears to be declining among individuals with AHI in Bangkok and in 2013 to 2014 met the World Health Organization definition for low prevalence. Continued surveillance is necessary to determine if this trend persists.

## Introduction

Men who have sex with men (MSM) are the largest single group acquiring new HIV infections in Thailand, accounting for over 40% of new infections in 2013 [1]. Despite an overall decrease in new HIV infections each year, and decreasing incidence among heterosexuals and people who inject drugs, the annual number of new HIV infections among MSM in Thailand continues to rise [2]. For the past decade, the HIV prevalence among MSM in Bangkok has remained 20 to 30% [3], and HIV incidence was consistently above 5 per 100 person-years [4–6]. At the Thai Red Cross Anonymous Clinic, the largest HIV testing centre in the country, 92% of acute HIV infections (AHI) identified are in MSM [7].

AHI presents the most opportune time to test for transmitted drug resistance (TDR). Previous research has shown that TDR is found at higher prevalence during AHI than in chronically infected patients [8], and that resistance mutations detected early in HIV infection may eventually become undetectable in the absence of selective pressure from antiretroviral therapy (ART) [9,10]. Such archived but undetectable mutations can lead to early treatment failure after initiation of ART [11,12].

TDR prevalence varies greatly by geographic region and population studied. A recent meta-analysis reported 2.9% TDR overall in South and Southeast Asia with no significant trend over time [13]. By comparison, TDR prevalence was 9.4% in Europe and 11.5% in North America. In Bangkok, TDR has been reported at 4.0 to 4.9% [14,15]. Among Chinese MSM across 19 provinces, TDR was 4.9% [16]. Cohorts with a majority of MSM in the high-income countries of Japan and Taiwan reported TDR prevalence of 9.1 and 11.1%, respectively [17,18]. The World Health Organization (WHO) classifies the level of TDR as low (<5%), moderate (5–15%) and high (>15%) [19].

We have previously reported a 9.2% prevalence of TDR in a Thai cohort that comprised primarily of MSM with AHI [20]. In this analysis, we report on longitudinal data from the same cohort and assess the change in TDR prevalence over time.

## Methods

The SEARCH 010/RV 254 study (clinicaltrials.gov NCT00796146) is an ongoing prospective, a longitudinal cohort study of participants with AHI in Bangkok and Pattaya, Thailand. The methods for identifying and enrolling individuals with protocol-defined AHI have been described previously [7,21]. Briefly, subjects are identified with AHI at selected HIV testing centres if they have a non-reactive fourth generation (4thG) enzyme immunoassay (EIA) detecting HIV antigen and HIV antibodies and positive HIV nucleic acid testing (NAT), or a reactive 4thG EIA and are non-reactive on a less sensitive second generation (2ndG) EIA detecting only anti-HIV IgG. Subjects are staged according to the published 4thG AHI staging system: stage 1 (NAT+, 4thG EIA-, third generation [3rdG] EIA-), stage 2 (NAT+, 4thG EIA+, 3rdG EIA-) and stage 3 (NAT+, 4thG EIA+, 3rdG EIA+, Western Blot- or indeterminate) [21]. Subjects presenting for HIV screening between 20 April 2009 and 31 December 2014 were included in this analysis. All subjects in the cohort were offered ART regardless of CD4 count using drug regimens recommended in the Thai National HIV Treatment guidelines [22]. ART regimens were adjusted if any resistance mutations were discovered on genotype testing. All subjects provided written informed consent to participate in the study. The study protocol was approved by the institutional review boards of Chulalongkorn University, Bangkok, Thailand, and the Walter Reed Army Institute of Research, Silver Spring, MD, USA.

### HIV genotyping

HIV genotyping was performed to detect mutations in the reverse transcriptase (RT) and protease (PR) genes at the time of enrolment into the cohort, before the initiation of ART. The TRUGENE HIV-1 genotyping assay (Siemens Healthcare Diagnostics, Bayswater, Victoria, Australia) was used for the first 66 samples, and a validated in-house method was used for the remaining samples [23]. Genotype sequences were analyzed using the Stanford University Drug Resistance Database [24,25], and mutations were categorized according to the WHO surveillance drug resistance mutation (SDRM) list [26]. Each mutation was classified as conferring resistance to nucleoside and nucleotide analogue reverse transcriptase inhibitors (NRTI), non-nucleoside analogue reverse transcriptase inhibitors (NNRTI) or protease inhibitors (PI). HIV subtype was screened using the multiregion hybridization assay (MHAbce) [27] for the first 63 samples and HIVSeq [28,29] for the remaining samples. HIV-1 subtype was determined using NCBI Genotyping tool (www.ncbi.nlm.nih.gov/projects/genotyping/formpage.cgi) and jpHMM tool (www.jphmm.gobics.de). Molecular Evolutionary Genetics Analysis (MEGA) 5.0 was used to construct a maximum likelihood tree of the sequences from this study and reference HIV-1 subtype strains to further confirm genetic subtypes.

### Statistical analysis

Prevalence of TDR was calculated by dividing the number of subjects with detectable SDRM by the total number of subjects with available genotyping data. Ninety-five percent confidence intervals (CIs) were calculated using the Clopper-Pearson interval. Trend over time was assessed using the Chi-squared test for trend. Because of the small number of subjects enrolled annually early in the study, data were analyzed using two-year intervals from 2009 to 2014. All analyses were performed using Stata Statistical Software Release 13 (Stata Corp, College Station, TX, USA). Significance was set at alpha equal to 0.05, and all *p*-values are two-sided.

## Results

From April 2009 to December 2014, the study enrolled 233 subjects with AHI. Genotype test results were available for 229 subjects, of whom 228 had successful sequencing of the RT gene and 225 had sequencing of the PR gene. Two subjects did not have adequate sample for genotype testing, and two samples failed to amplify. Characteristics of the study population are reported in Table 1. Median age was 28 years, 95% were male and 92% were MSM. The median time from HIV exposure to study entry was 18 days. Median HIV RNA viral load was 5.8 log_10_copies/ml. The most common HIV subtype was CRF01_AE (80.6%). Results of fourth generation staging are shown in Table 1, with no significant change in stage at enrolment over time (data not shown).

**Table 1:** Characteristics of the population with acute HIV infection in Bangkok, Thailand

Characteristics	(*n*=229)
Age, years, median (IQR)	28 (23–32)
Male, *n* (%)	218 (95)
Sexual orientation, *n* (%)	
MSM	212 (93)
Heterosexual male	6 (2)
Heterosexual female	11 (5)
Duration since history of HIV exposure, median (IQR) days	18 (14–24)
Sexual partners in the past 30 days, median (range)	2 (1–20)
Recreational drug use in the past 30 days, *n* (%)	51 (22)
4th G staging	
Stage 1 (NAT+, 4thG EIA−, 3rdG EIA −)	31 (14)
Stage 2 (NAT+, 4thG EIA+, 3rdG EIA −)	71 (31)
Stage 3 (NAT+, 4thG EIA+, 3rdG EIA+, WB–/indeterminate)	127 (55)
HIV RNA (log10 copies/mL), Median (IQR)	5.8 (5.2–6.8)
CD4 (cells/mm^3^), Median (IQR)	354 (265–490)
Subtype	
CRF01_AE	183 (80.6)
B	10 (4.4)
C	1 (0.4)
01AE/B	25 (11.0)
01AE/B/C	1 (0.4)
01AE/02AG	1 (0.4)
Non-typable	6 (2.6)

IQR, inter-quartile range; MSM, men who have sex with men; NAT, nucleic acid testing; EIA, enzyme immunoassay; WB, western blot.

**Figure 1:**
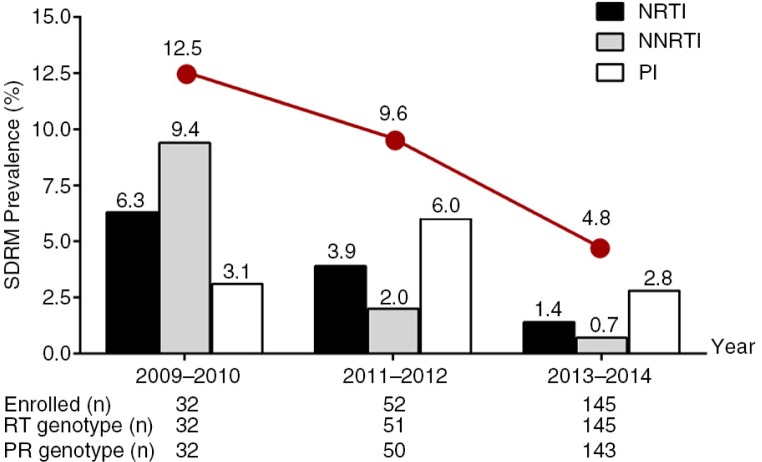
Change over time in surveillance drug resistance mutation (SDRM) prevalence among patients with acute HIV infection in Bangkok, Thailand (*n*=229). Bars represent prevalence by drug class; the line represents the total prevalence for SDRM in the cohort.

Mutations from the WHO SDRM list were identified in 16 subjects for an overall TDR prevalence in the cohort of 7.0% (95% CI: 4.0–11.1%) (Table 2). Prevalence of resistance by drug class was highest for the PI drugs at 3.6% (1.5–6.9%), followed by NRTI 2.6% (1.0–5.6%) and NNRTI 2.2% (0.7–5.0%). TDR prevalence declined from 12.5% in 2009–2010 to 4.8% in 2013–2014 (Figure 1), although the trend was not statistically significant (*p*=0.08). There was a significant decline in resistance to NNRTI from 9.4% in 2009–2010 to 0.7% in 2013–2014 (*p*=0.01). NRTI resistance demonstrated a non-significant decline from 6.3 to 1.4% (*p*=0.09), whereas resistance to PI had no discernible trend over time (*p*=0.64).

**Table 2:** SDRM prevalence by year

	Overall	2009–2010	2011–2012	2013–2014	*p*
*N* enrolled	229	32	52	145	–
Any resistance, *n* (%)	16 (7.0)	4 (12.5)	5 (9.6)	7 (4.8)	0.08
*N* with RT genotype	228	32	51	145	–
NRTI	6 (2.6)	2 (6.3)	2 (3.9)	2 (1.4)	0.09
NNRTI	5 (2.2)	3 (9.4)	1 (2.0)	1 (0.7)	0.01
*N* with PR genotype	225	32	50	143	–
PI	8 (3.6)	1 (3.1)	3 (6.0)	4 (2.8)	0.64

RT, reverse transcriptase; NRTI, nucleoside/nucleotide reverse transcriptase inhibitor; NNRTI, non-nucleoside reverse transcriptase inhibitor; PR, protease region; PI, protease inhibitor.

The most common SDRM were K103N and M46I, each found in three participants. The M41L and Y181C mutations were each found in two participants. Three additional subjects had mutations at the E138 codon in the RT gene (E138A in two subjects and E138K in one subject), which confers low-level resistance to the NNRTI class and is included in the IAS-USA list of HIV resistance mutations [30], but does not appear in the WHO SDRM list. All SDRM detected and a summary of the characteristics of the 16 subjects with SDRM are listed in Table 3.

**Table 3:** Characteristics of acute HIV-positive patients with transmitted drug resistance mutations

ID	Date^a^	Age	Risk	Days since HIV exposure	Fiebig stage	4thG stage	HIV RNA^b^	CD4^c^	HIV subtype	Resistance mutations		

										NRTI	NNRTI	PI
01	05/09	28	MSM	14	2	2	6.5	426	CRF01_AE	–	K103N	–
02	06/09	29	MSM	20	3	3	5.5	740	CRF01_AE	–	K103N	–
03	03/10	34	MSM	15	3	3	5.6	386	CRF01_AE	T215F	Y181C	M46I
04	08/10	24	MSM	24	5	3	5.5	736	CRF01_AE	M41L	–	–
05	07/11	26	HF	17	1	2	5.8	342	CRF01_AE	–	G190A, Y181C	–
06	02/12	30	MSM	13	3	3	7.5	295	CRF01_AE	–	–	F53Y
07	03/12	23	MSM	32	3	3	5.6	506	CRF01_AE	–	–	L23I
08	11/12	34	MSM	8	1	1	4.4	878	CRF01_AE	D67N	–	V82A
09	12/12	45	MSM	19	1	2	5.6	303	B	T215E	–	–
10	07/13	22	MSM	20	1	1	3.6	519	CRF01_AE	L74I	–	–
11	08/13	20	MSM	24	1	1	4.1	332	CRF01_AE	M41L	–	–
12	01/14	31	MSM	15	1	2	5.5	709	CRF01_AE/B	–	–	M46I
13	06/14	43	MSM	20	3	3	5.7	433	CRF01_AE	–	–	M46I
14	07/14	26	MSM	3	3	3	5.8	602	CRF01_AE	–	–	G73S
15	11/14	19	MSM	20	3	3	7.0	222	CRF01_AE	–	–	M46L
16	12/14	28	MSM	26	3	3	6.4	545	CRF01_AE	–	K103N	–

MSM: Men who have sex with men; HF: heterosexual female.

a Month/year of study enrolment

b log10 copies/ml

c cells/mm^3^

One study participant had TDR to two drug classes (NRTI and PI), and another study participant had TDR to all three drug classes. One pair, subjects 1 and 2, were sexual partners with linked HIV transmission by reported history and an identical resistance mutation on genotype testing. Full-length sequencing showed a 0.1% genetic difference in the two viral genomes, phylogenetically confirming the linked HIV transmission. Using a genetic distance of 1.5% as a cut-off, no other linked transmission pairs were found among participants with TDR.

## Discussion

We observed a declining trend in overall TDR prevalence in this prospective cohort of AHI in Thailand. The change was most evident in resistance to NNRTI, which showed a statistically significant decrease from 9.4 to 0.7% over time. NRTI resistance declined from 6.3 to 1.4%, although the change was not statistically significant, while PI resistance had no discernible trend. The low and declining rate of NNRTI resistance is reassuring, given that the current preferred first-line ART regimens in both the Thai and WHO HIV treatment guidelines contain the NNRTI efavirenz [22,31].

The 4.8% prevalence of TDR in this cohort in 2013 to 2014 was similar to other reports from Thailand, which have found TDR prevalences of less than 5% [14,15]. A recent meta-analysis also reported a pooled TDR prevalence of 2.9% from seven low- and middle-income countries in South and Southeast Asia [13]. Several studies of MSM cohorts in China have reported similar TDR prevalences of 4.6 to 5.3% [16,32].

A few studies have reported that MSM in Asia have higher TDR prevalence than heterosexuals [33,34]. However, it remains unknown whether this finding is because of a true difference in TDR between MSM and other transmission risk groups, or if it is an artefact of the evolving HIV epidemiology in Asia. MSM are more likely to be recently infected at the time of HIV diagnosis, and TDR detected in early infection may become undetectable by standard assays over time [2,9,10].

It is not clear why TDR would be declining over time in this cohort, but changes in ART regimens used in public programmes over time could theoretically contribute to better drug adherence and decreased resistance in treated populations. Consistent with the WHO guidelines and as in many other developing countries, in recent years Thailand has changed the preferred first-line regimen for HIV treatment to drugs that require less frequent dosing, have fewer side effects and are easier tolerated by patients, all characteristics that have been associated with better treatment adherence [35]. The Thai treatment guidelines prior to 2010 included stavudine, zidovudine and nevirapine in first-line regimens; all of which have significant risk of toxicity and require twice-daily dosing [22,36]. The current Thai guidelines recommend tenofovir, efavirenz and lamivudine or emtricitabine as the preferred first-line regimen for all patients, which can be dosed once-daily and has fewer side effects than the older regimens [36]. Better adherence among people on ART could directly lead to less treatment failure, fewer people with resistance mutations and less TDR in the community.

The patients in this cohort came from a limited number of sites in Bangkok and Pattaya, Thailand, which may limit the generalizability of the findings to other locations. The small sample size, particularly in the first two years of the cohort, may also affect the accuracy of the TDR prevalence estimate for that time period. In addition, the AHI cohort is predominantly MSM; populations with other risk factors for HIV transmission may have different patterns of TDR.

The major advantage of this study is that genotype resistance testing was performed very early in HIV infection, a median of 18 days after HIV exposure. Most other studies of TDR enrol patients in the chronic phase of HIV infection, when genotype testing is less sensitive for the detection of drug resistance [8–10].

## Conclusions

We found a declining trend in TDR among MSM with recently acquired HIV infection in Thailand, with TDR prevalence of 4.8% in 2013 to 2014. Prevalence below 5% meets the WHO definition of low level TDR, where routine genotype testing for resistance prior to the initiation of first-line ART is not recommended [19]. However, continued surveillance of TDR should be performed to monitor for any change as ART use expands and the HIV epidemic in Thailand continues to evolve.
